# The Treatment of Acquired Hemophilia with Combination Therapy of Immunosuppressives and Immunoadsorption

**DOI:** 10.4274/tjh.2013.0178

**Published:** 2014-06-10

**Authors:** Aynur Uğur Bilgin, Muhit Özcan, Erol Ayyıldız, Osman İlhan

**Affiliations:** 1 Necmettin Erbakan Universty Meram Faculty of Medicine, Department of Hematology, Konya, Turkey; 2 Ankara University Faculty of Medicine, Department of Hematology, Ankara, Turkey

**Keywords:** Factor VIII deficiency, Acquired, Immunosuppressive

## TO THE EDITOR

Acquired hemophilia A (AHA) is an uncommon entity caused by autoantibodies against blood clotting factor VIII, which neutralize its procoagulant activity. The incidence of AHA is approximately 1 to 4 per million/year [1] and the underlying pathological conditions of the disease are still unknown. Although the majority of patients with acquired hemophilia are idiopathic (up to 50%), there are possible associated illnesses such as autoimmune disease, solid tumors, lymphoproliferative malignancies, skin disorders, drugs, infections, and chronic graft-versus-host disease, and AHA occurs most commonly in the elderly [2]. We report here an AHA patient who was successfully treated with a combination therapy of immunosuppression and immunoadsorption.

A 78-year-old male patient, who had symptoms including epistaxis, spontaneous ecchymosis, and hematomas on the back and hip, referred to our hospital in March 2010. His medical history included chronic obstructive pulmonary disease and he had no history of preexisting autoimmune disease or inherited hemorrhagic disorders. On physical examination, multiple ecchymosis and hematomas were present on his left shoulder, arm, and thigh. When auscultating the lungs of the patient, wheezes were bilaterally heard over the lower lung fields. No other abnormality was noticed on physical examination. The initial laboratory findings showed a white blood cell count of 12.3x10^9^/L (normal range: 4.5-11x10^9^/L) with neutrophils at 84%, hemoglobin of 7.3 g/dL (normal range: 12.6-17.4 g/dL), hematocrit of 22.1% (normal range: 37%-51%), and platelets of 430x10^9^/L (normal range: 150-400x10^9^/L). Biochemical parameters were normal; however, coagulation studies showed that the normal prothrombin time and international normalized ratio, which were 12.6 s and 1.09, respectively, and prolonged activated partial thromboplastin time [aPTT: 157 s (normal range: 22-36 s)] were not corrected by a 1:1 mixture with normal fresh plasma after a 2-h incubation period. Informed consent was obtained.

The anticoagulant effect of the patient was not corrected by dilution, suggesting the presence of an inhibitor directed against one of the coagulation factors. Additionally, the factor VIII:C level and its inhibitor titer were determined as 3.9% and 9.6 BU, respectively. The patient was diagnosed with AHA. Etiological diagnostic workup including autoimmune and tumor markers was negative. Thorax and abdominopelvic computed tomography revealed no pathological findings. Following the transfusion of erythrocyte suspensions, the patient was treated with recombinant FVIIa (rFVIIa Novo Seven®) at a dosage of 90 µg/kg (3 times) to control the Hemorrhagic syndrome. After the recombinant FVIIa treatment, bleeding was controlled. Although there was a slight improvement in his laboratory values, aPTT never returned to the normal range. The factor VIII:C level and its inhibitor titer reached a value of 0.4% and 44 BU, respectively, 1 month later. Cyclophosphamide at 50 mg/day plus methyl-prednisolone (MP) at 1 µm/kg/day was initiated as immunosuppressive therapy. However, there was no improvement in factor VIII:C level and its inhibitor titer (2% and 52.8 BU, respectively) after 1 month from the beginning of immunosuppressive drugs, and his complaints about nose bleeding and spontaneous ecchymoses on multiple areas of his body began again. In order to stop bleeding complications, recombinant activated factor VII (rFVIIa) at a dose of 90 µg/kg (3 times) was given again. We decided to administer a modified Bonn-Malmö Protocol [large-volume immunoadsorption on days 1-5, intravenous immunoglobulin 0.3 g/kg/day on days 5-7, cyclophosphamide 1 mg/kg/day orally, MP 1 mg/kg/day orally from day 1 until remission (dose reduction) as immunosuppressive therapy, and factor VIII 100 IU/kg every 6 h (dose reduced when 50%-80% FVIII activity was achieved)] as described before [2]. After 5 courses of immunoadsorption procedure, laboratory findings showed aPTT of 31.3 s, factor VIII:C level of 81.7%, and factor inhibitor of 0. As a result, cyclophosphamide was not given anymore, and MP was tapered toward an end. The patient was discharged with MP alone as immunosuppressive therapy. One month after the therapy, laboratory results of the patient were normal and no bleeding complications occurred. Unfortunately, we learned from the patient’s relatives that he died due to acute exacerbations of chronic obstructive pulmonary disease at 6 months.

AHA is a rare but potentially fatal condition that can lead to life-threatening hemorrhage. The bleeding phenotype is heterogeneous, but rather different from congenital hemophilia. Soft tissue hematoma, muscle bleeding, hematuria, gastrointestinal bleeding, and postpartum hemorrhages are typical clinical manifestations, as opposed to congenital hemophilia where hemarthroses are predominant [3]. The mortality rate of AHA ranges between 16% and 22% depending on age, underlying diseases, the levels of inhibitors, and individual response to therapy.

Because of the low incidence of AHA, therapeutic options of this condition are still discussed controversially and are not clearly standardized. Due to the high risk of life-threatening bleeding, an aggressive therapeutic approach is recommended in these patients. Currently 2 important therapeutic strategies are the treatments of acute bleeding: high doses of factor VIII concentrates and/or factor VIII bypassing agents (activated prothrombin complex concentrate or recombinant factor VIIa) and the eradication of the factor VIII autoantibodies from plasma [by immunosuppressive drugs (the combination of steroids and cyclophosphamide) and/or plasmapheresis or immunoadsorption] [1,2,3]. Immunoadsorption is a treatment that can be performed with diverse extracorporeal systems and it depletes the IgG fraction from the plasma of patient. Compared to plasmapheresis, larger volumes of plasma are processed and the procedure is more efficient in immunoadsorption. There are some case series that demonstrate the efficacy of immunoadsorption in acquired hemophilia [4,5,6,7]. Xu et al. presented a 58-year-old man with AHA treated with a combination of low-dose rituximab and recombinant human FVIIa, and they suggested that rituximab therapy should be considered for inhibitor eradication treatment of AHA [8].

In our case, immunosuppressive treatment, consisting of MP and cyclophosphamide, was administered as the first-line therapy. However, neither a clinical nor a laboratory response was observed. We thus made a decision to apply a modified Bonn-Malmö Protocol [1] to the patient. Immediately after applying this protocol, from the fifth day on, the patient’s laboratory results returned to normal and clinical bleeding ended. Therefore, this case showed that immunoadsorption procedure added to immunosuppressive medication is an efficient treatment modality for AHA. In conclusion, the application of a modified Bonn-Malmö Protocol is easy, reliable, and safe, and it can be considered as the first line of treatment, especially in elderly patients. 

## CONFLICT OF INTEREST STATEMENT

The authors of this paper have no conflicts of interest, including specific financial interests, relationships, and/ or affiliations relevant to the subject matter or materials included.
